# Perioperative anesthetic management of intestinal pseudo-obstruction as a complication of pheochromocytoma

**DOI:** 10.1186/s40981-019-0255-9

**Published:** 2019-05-23

**Authors:** Saki Okumura, Makoto Sumie, Yuji Karashima

**Affiliations:** 1 Department of Anesthesiology and Critical Care Medicine, Kyushu University Hospital, Fukuoka, Japan; 2 Operating Rooms, Kyushu University Hospital, Fukuoka, Japan; 3 Department of Anesthesiology and Critical Care Medicine, Graduate School of Medical Sciences, Kyushu University, Fukuoka, Japan

**Keywords:** Pheochromocytoma, Intestinal pseudo-obstruction, α-Adrenergic receptor blocker, Phentolamine, Epidural anesthesia

## Abstract

**Background:**

Intestinal pseudo-obstruction, which is a rare complication of pheochromocytoma, can be caused by hypersecreted catecholamines.

**Case presentation:**

A 45-year-old woman was admitted for local recurrence of pheochromocytoma complicated by intestinal pseudo-obstruction. The intestinal pseudo-obstruction showed poor response to α-adrenergic receptor blocker and she was scheduled for surgical resection of pheochromocytoma. The surgery was uneventfully accomplished with general anesthesia combined with epidural anesthesia. The latter was performed with the aim of not only perioperative pain management but also of promoting intestinal peristalsis. The anticipated effect for intestinal peristalsis was not apparent in the early postoperative phase. The abdominal symptoms were gradually relieved over the course of about 1 month.

**Conclusions:**

For intestinal pseudo-obstruction induced by pheochromocytoma, although inhibition of the sympathetic system by epidural infusion of local anesthetics may be promising, short-term usage of epidural local anesthetics infusion did not provide a quick recovery after pheochromocytoma removal surgery.

## Introduction

Gastrointestinal pseudo-obstruction, or paralytic ileus, can be caused by pheochromocytoma with hypersecretion of catecholamines, which act on α_2_-adrenergic receptors of intestinal smooth muscle cells to decrease intestinal peristalsis [1, 2]. Although several cases have been reported, the literature contains few descriptions of perioperative anesthetic management for these patients [3–6]. Because of the absence of any obstructive structural lesions in the intestinal tract, we presumed that a strategy for promoting intestinal peristalsis postoperatively would be advantageous. In this report, we focus on perioperative anesthetic management of intestinal pseudo-obstruction complicated with pheochromocytoma.

## Case

**Fig. 1:**
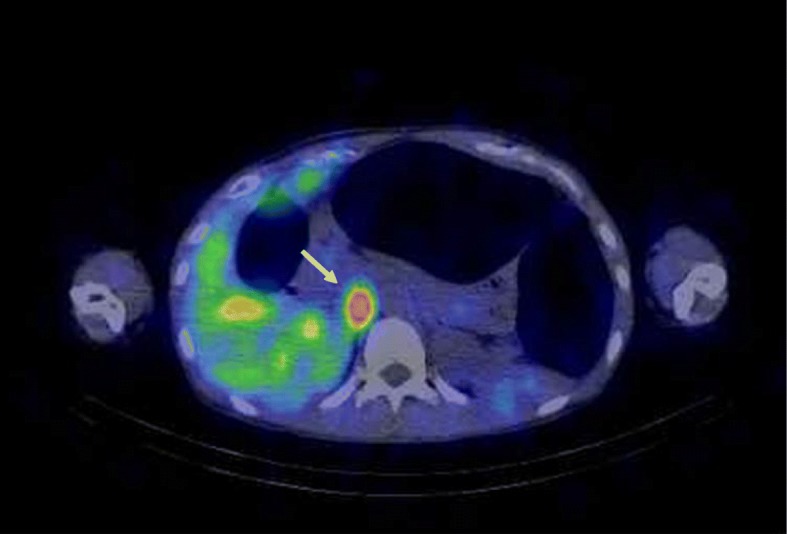
^123^I-metaiodobenzylguanidine scintigraphy shows a 17 × 15 mm mass accumulation on the left of the inferior vena cava (white arrows)

**Fig. 2:**
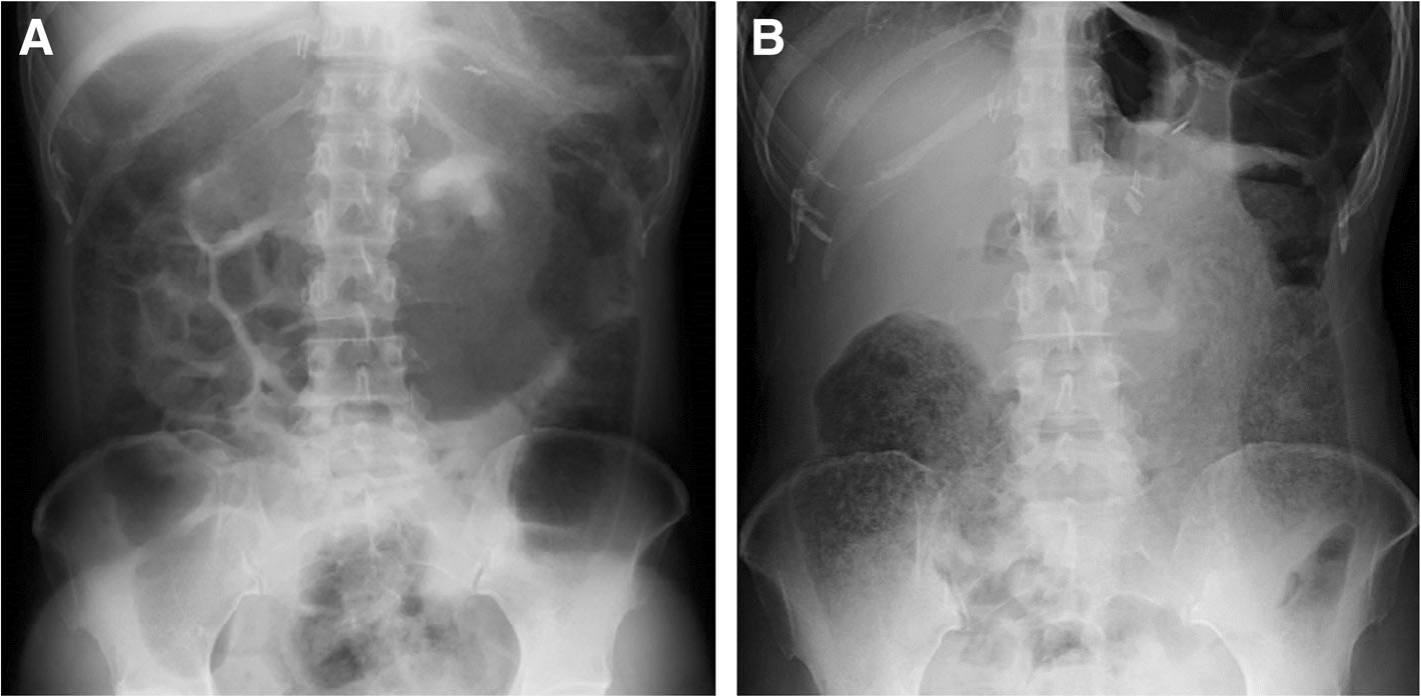
Abdominal radiograph on admission (**a**) and on postoperative day 30 (**b**). Remarkable distended bowel and gas on admission was relieved on postoperative day 30

A 45-year-old-woman (height 162.9 cm, weight 44.5 kg) was admitted with complaints of twice-daily headaches, abdominal pain, nausea, obstipation over several days, and excretion of only a small amount of aqueous stool in spite of enema administrations. She had a medical history of lip mucosal neuroma resection at the age of 6, total thyroidectomy for medullary carcinoma at the age of 14, and right and left adrenalectomy for pheochromocytoma at the ages of 25 and 27, respectively. She had received follow-up on suspicion of multiple endocrine neoplasia type 2B. Physical examination revealed that the abdomen was soft but bulging, with weakened intestinal peristaltic sound. Blood pressure was 110–150/60–90 mmHg, heart rate was 90–120 beats/min in sinus rhythm, and body temperature was 36.8 °C. Subsequent 24-h urine collection for catecholamines revealed elevated metanephrine levels of 0.4 [normal range (NR) 0.04–0.19] μg/day, normetanephrine levels of 0.4 (NR 0.09–0.33) μg/day, dopamine levels of 3241.8 (NR 365.0–961.5) μg/day, adrenaline levels of 40.7 (NR 3.4–26.9) mg/day, and noradrenaline levels of 253.1 (NR 48.6–168.4) mg/day. ^123^I-metaiodobenzylguanidine (MIBG) scintigraphy showed a 17 × 15 mm mass accumulation on the left side of the inferior vena cava (Fig. 1). X-ray and computed tomography of the abdomen showed remarkably distended colon with the maximal diameter of 12.6 cm and colon gas, which were consistent with the patient’s symptoms (Fig. 2a). She was diagnosed with local recurrence of pheochromocytoma and scheduled for laparoscopic tumor resection. For preoperative preparation, several strategies for reduction of intra-intestinal pressure were initiated, which included fasting, 2000 ml/day of fluid replacement, oral administration of α_1_-adrenergic receptor blocker, doxazosin (1 mg/day), and intravenous administration of the nonselective α-adrenergic receptor blocker phentolamine (1.5 mg/h). Furthermore, lower intestinal endoscopy showed a large amount of fecal mass and enema were performed. However, despite all those interventions, defecation was difficult and neither intra-intestinal pressure reduction nor symptomatic relief was achieved.

We selected general anesthesia combined with epidural anesthesia for perioperative management of this patient. An epidural catheter was placed between the ninth and tenth thoracic vertebra, and its efficacy was confirmed by administration of a combination of 8 ml of 2% mepivacaine and 100 μg of fentanyl before induction of anesthesia. In addition to standard monitors, invasive arterial pressure via radial artery and stroke volume index (SVI) and stroke volume variation (SVV) with the use of the FloTrac™-EV1000™ system (Edwards Lifesciences, Irvine, CA, USA) were monitored. The induction was uneventful with 2 μg/kg of fentanyl, 2 mg/kg of propofol, and 0.6 mg/kg of rocuronium under reverse Trendelenburg position and pressured cricothyroid cartilage. Anesthesia was maintained with desflurane (0.5–0.7 MAC), remifentanil (0.15–0.4 μg/kg/min), and 0.25% levobupivacaine (5 ml/h) via epidural catheter. Although hemodynamic fluctuations occurred during manipulation of the tumor, the patient was successfully managed with bolus infusions of phenylephrine (0.1 mg), nicardipine (0.2 mg), and landiolol (2.5 mg); neither of continuous infusion of vasodilators nor catecholamines was needed. Intraoperative fluid infusion was titrated to maintain SVI values between 40 and 80 ml/m^2^/beat and SVV between 5 and 10%. For postoperative analgesia, intravenous acetaminophen (700 mg) was administered and continuous epidural infusion of 0.125% levobupivacaine (5 ml/h) was initiated at the end of surgery.

Postoperative pain was well controlled with continuous epidural infusion of local anesthetics and regular administration of acetaminophen. On the second postoperative day, the numerical pain rating scale was almost 1 and the epidural catheter was removed. Acetaminophen was continued postoperatively for 1 week. Regarding the patient’s postoperative symptoms, although headache and nausea were gradually relieved, intestinal pseudo-obstruction and defecation difficulties continued over several weeks postoperatively (Fig. 2b), which required several stool suction treatments by lower gastrointestinal endoscopy. Finally, the patient was able to resume oral intake on the 22nd postoperative day.

## Discussion

Pheochromocytomas are rare neuroendocrine tumors secreting high levels of catecholamines that produce highly variable clinical signs and symptoms. Although hypertension, tachycardia, pallor, headache, and anxiety usually dominate the clinical presentations, gastrointestinal manifestations, such as nausea, abdominal pain, and chronic constipation, are also reported in 20–40% of patients with pheochromocytoma [1, 2]. Gastrointestinal pseudo-obstruction, which differs from mechanical ileus in that there is no demonstrable blocking lesion, is a rare and relatively unknown but potentially life-threatening complication of pheochromocytoma. Osinga et al. report, in a review that describes 34 cases of pseudo-obstruction associated with pheochromocytoma and paraganglioma in 32 publications, that 5 patients (15%) developed bowel perforation and 16 patients (47%) died within 1 year after the development of the condition [7]. Its pathophysiology can be a direct consequence of the elevated catecholamine levels that reduce the peristalsis of the gastrointestinal tract. Gastrointestinal activity is controlled by the parasympathetic and sympathetic nervous systems. The activation of the parasympathetic nervous system causes acetylcholine release, which stimulates muscarinic receptors and leads to bowel movement. In contrast, activation of the sympathetic nervous system releases noradrenaline, which inhibits bowel motility through activation of the α_1_-, α_2_-, and β_2_-adrenergic receptors. The activity of the sympathetic nervous system is further augmented by a presynaptic noradrenaline-mediated inhibition of parasympathetic acetylcholine release. In addition, circulating catecholamines can not only inhibit gastrointestinal peristaltic activity through stimulation of β_2_ receptors but also cause intestinal vasoconstriction and ischemia through stimulation of α_1_ and α_2_ receptors [4, 8, 9].

The first-line treatment for pheochromocytoma-associated gastrointestinal pseudo-obstruction is a conservative approach, which includes fasting, nasogastric suction, and intravenous replacement of fluids and electrolytes. If initial treatment is not successful, endoscopic desufflation and pharmacological treatment should be considered. Some reports suggest the use of nonselective α blockers, such as phentolamine or phenoxybenzamine, for the treatment of gastrointestinal complications of pheochromocytoma [7]. However, neither phentolamine nor doxazosin was effective for our patient. The ultimate therapy is surgical removal of the pheochromocytoma. Although there are some reports about surgical treatment, anesthetic considerations are scarcely described, and as far as we know, this is the first description of the anesthetic management of gastrointestinal pseudo-obstruction as a complication of pheochromocytoma.

From the pathophysiological characteristics of gastrointestinal pseudo-obstruction, we reasoned that inhibition of the gastrointestinal sympathetic system perioperatively by epidural infusion of local anesthetics would be advantageous for the patient to promote intestinal peristalsis. In addition, epidural analgesia would be beneficial in terms of avoiding postoperative opioid administration, which inhibits intestinal peristalsis by activating intestinal μ receptors [9]. Although the postoperative pain of our patient was well-controlled without using opioids, the anticipated effect of improved bowel movement was not apparent. One of the reasons for this could be attributed to the very short duration of epidural infusion of local anesthetics postoperatively. However, on the grounds that many factors, e.g., perioperative fluid infusion and inflammation, can influence bowel movement in an early postoperative period, it is impossible to determine whether the epidural infusion had little effect on our patient or the other factors overwhelmed it. Longer use of epidural infusion might have had an effect on intestinal peristalsis; however, we have no evidence to prove it. Another possibility can be maldistribution of adrenergic receptors, which should be affected by over-secreted catecholamines due to pheochromocytoma and would recover gradually postoperatively, which might be consistent with our patient’s recovery time course. But again, we have no way to prove it.

In conclusion, this case report illustrates the anesthetic consideration of intestinal pseudo-obstruction induced by pheochromocytoma. From a pathophysiological point of view, epidural anesthesia may be promising in terms of promoting intestinal peristalsis postoperatively by inhibiting the sympathetic system; however, in our patient, a beneficial effect was not apparent.

## Data Availability

Please contact the corresponding author for data requests.

## Abbreviations

MIBG: ^123^I-metaiodobenzylguanidine
NR: Normal range
SVI: Stroke volume index
SVV: Stroke volume variation
